# Can Merging the Roles of Public Health Preparedness and Emergency Management Increase the Efficiency and Effectiveness of Emergency Planning and Response?

**DOI:** 10.3390/ijerph110302911

**Published:** 2014-03-10

**Authors:** Nadja A. Vielot, Jennifer A. Horney

**Affiliations:** 1 Department of Epidemiology, Gillings School of Global Public Health, University of North Carolina at Chapel Hill, CB 7435, Chapel Hill, NC 27599, USA; E-Mail: nadjavielot@unc.edu; 2 Department of Epidemiology and Biostatistics, School of Rural Public Health, Texas A&M University, 1266 TAMU, College Station, TX 77843, USA

**Keywords:** emergency preparedness, emergency response, planning

## Abstract

Some jurisdictions have reduced workforce and reallocated responsibilities for public health preparedness and emergency management to more efficiently use resources and improve planning and response. Key informant interviews were conducted in six counties in North Carolina (USA) to discuss perceptions of the challenges and opportunities provided by the new shared positions. Respondents feel that planning and response have improved, but that requirements related to activities or equipment that are eligible for funding (particularly on the public health side) can present an impediment to consolidating public health preparedness and emergency management roles. As the financial resources available for public health preparedness and emergency management continue to be reduced, the merging of the roles and responsibilities of public health preparedness and emergency management may present jurisdictions with an effective alternative to reducing staff, and potentially, readiness.

## 1. Introduction

In the wake of disasters, various public health, emergency response, and government agencies must work together to implement preparedness plans and mount an effective response that may include activities as varied as opening and operating shelters, rapidly assessing unmet needs for utilities and other essential services, and providing services such as debris removal to residents. Since 2002, federal funding for public health preparedness has been provided by the Centers for Disease Control and Prevention (CDC) to state, local, tribal, and territorial public health departments through the Public Health Emergency Preparedness (PHEP) cooperative agreement. PHEP cooperative agreements are the main source of funding local health departments (LHDs) use to develop and maintain their ability to effectively respond to public health threats, including infectious diseases, natural disasters, and biological, chemical, nuclear, and radiological events [1]. Many states have used PHEP funds to fund positions for public health preparedness coordinators (PCs). These PCs lead the LHD’s efforts related to planning, exercising, and working with partners and volunteers that are necessary to improve the capacity of LHDs to plan for, respond to, and recover from public health emergencies.

In recent years, PHEP funding has declined significantly. From fiscal years 2005–2012, federal preparedness funds to state and local health departments from CDC were reduced more than 38% (adjusted for inflation) [2] Economic surveillance surveys conducted by the National Association of County and City Health Officials (NACCHO) from 2008–2011 found that many LHDs have experienced budget cuts, loss of staff, and service reductions [3]. In 2011, 23% of LHDs surveyed by NACCHO reported that they had reduced or eliminated their emergency preparedness program, making it the program area, along with clinical health services, most frequently cut [3]. At the same time, the economic recession has negatively impacted funding for emergency management. The National Emergency Management Association estimates that more than a dozen states have eliminated positions in emergency management or furloughed employees, negatively affecting planning, training, and exercises [4]. Perhaps more critically, nearly 60% of states report difficulties in meeting the 50% match required for their state to receive funding from the Emergency Management Performance Grant, the only source of federal money directed to state and local governments for planning, training, exercises, and personnel for all-hazards emergency preparedness [4].

Historically, emergency managers have operated under the “command and control” paradigm, in which response activities were mandated by governments and executed by local law enforcement, fire departments, and emergency medical services [5,6]. Emergency managers were tasked with coordinating emergency operations of various responders, ensuring communications, and providing linkages to policy makers [7]. More recently, emergency management has effectively transitioned to a focus on all hazards and comprehensive emergency management, which includes natural and technological disasters, as well as public health emergencies such as intentional events (e.g., the anthrax attacks of 2001) and naturally occurring pandemics (e.g., the 2009 novel influenza A (H1N1) pandemic). For example, principles for emergency management developed after the failed response to Hurricane Katrina in 2005 call for progressive, integrated, collaborative, coordinated, and flexible emergency managers [8]. In addition, the threat of bioterrorism has led to a greater appreciation of the importance of public health as first responders, and has increased coordination between public health and emergency response. Because public health organizations are well-engaged with their communities and are skilled in communications and outreach, disaster preparedness and response may be improved by combining the resources of public health with those of emergency management. As a result, emergency management and public health preparedness agencies and officials have increasingly been able to collaborate to develop emergency response protocols and promote community resilience [9]. 

In recent years, given the reduced threat of bioterrorism and increased threat of emerging infectious diseases (e.g., the 2009 novel influenza A (H1N1) pandemic), the importance of public health in emergency response has earned greater recognition and efforts to bridge the divide between emergency management and public health preparedness have increased. A schism still exists, however, between the two disciplines. Next steps in nurturing this relationship include the clarification of roles and responsibilities between county governments and health departments, and the development of preparedness plans that are clear, actionable, and proven through table-top exercises and simulations [10]. 

In light of recent budget cuts to LHDs and emergency management, some counties have been forced to reduce their workforce and reallocate responsibilities related to emergency preparedness and response. In six North Carolina counties, the role of public health preparedness coordinator has been merged with emergency management, emergency medical services, or hospital emergency management. To our knowledge, this experience is unique to these counties, and it is unclear how well this arrangement will affect emergency preparedness and response. To better understand the impact of combining these workforce assets in North Carolina, a study aimed at describing the roles and responsibilities of these merged positions was carried out by the North Carolina Preparedness and Emergency Response Research Center (NC PERRC) at the University of North Carolina at Chapel Hill Gillings School of Global Public Health. 

## 2. Experimental Section

Interviews were conducted with individuals performing roles that covered shared responsibilities in emergency management, emergency medical services, hospital preparedness, and public health preparedness. Three initial key informants (KIs) were identified through informal communication between the research team and attendees at the N.C. Division of Public Health’s Office of Public Health Preparedness and Response annual Preparedness Coordinator conference. Individuals initially contacted for interviews were then asked to identify others who might be working in the same or a similar capacity. Individuals were considered eligible to participate in the study if they reported having regular job responsibilities that included both emergency management, emergency medical services, or hospital-based emergency manager and public health preparedness coordination; represented a county of the state of North Carolina; and were based in a county government office, a county health department, or a county hospital system. 

**Figure 1:**
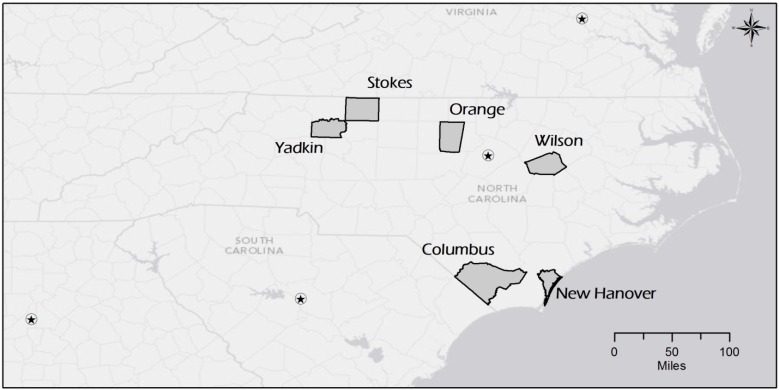
Map of Columbus, New Hanover, Orange, Stokes, Wilson, and Yadkin Counties, North Carolina, USA

Semi-structured phone interviews were conducted by a pair of interviewers with six KIs from the following counties in North Carolina: Columbus, New Hanover, Orange, Stokes, Wilson, and Yadkin (Figure 1). The interview guide was adapted from one used in a previous NC PERRC research project examining the structural capacity of Public Health Regional Surveillance Teams [11]. It included open-ended questions regarding the nature of the position in each county, the KIs opinions on the usefulness of the shared position, and their recommendations for implementing it in other counties. Closed ended questions asked KIs to identify key partners and resources among an exhaustive list of potential preparedness and emergency management partners. Where warranted, KIs were asked to provide examples of their experiences working under the new model during actual emergency responses or exercises to provide case study examples to inform other counties potentially interested in combining these positions in the future.

Interviews were recorded, transcribed, and analyzed to identify major themes. Each interview lasted between 30–60 min. Quotations that succinctly illustrated the perspective of a given KI were extracted and reprinted in the Results section. KIs are not identified by name, but by the county in which they are employed. The project was reviewed and determined exempt by the University of North Carolina at Chapel Hill Institutional Review Board (#14-0332).

## 3. Results and Discussion

### 3.1. Qualifications for Serving in Both the Emergency Management and Public Health Preparedness Capacities

Because the combined emergency management and public health preparedness coordinator position is relatively new, few formal requirements for the position were reported. Two KIs reported that Incident Command System (ICS) certification was required of applicants during the recruitment phase. Most respondents reported having a background in emergency management or emergency medical services; some had served as paramedics and firefighters in previous positions. No counties reported that public health credentials were required, though one reported that a registered nurse (RN) was preferred, but not required, for the position. 

### 3.2. Division of Responsibilities

In no case were the KIs’ responsibilities for emergency management and public health preparedness divided evenly. KIs from two counties reported that the majority of their time was devoted to emergency medical services and emergency management respectively, while those from two other counties reported that most of their time was dedicated to public health. Tasks and priorities differed depending on where KIs’ directives come from: emergency management or the local health department. The supervisory arrangements and scopes of work differed by county, depending on the unique needs of each:

“There’s been a large turnover in PC’s (Public Health Preparedness Coordinator) since I started in April (2013). And I’ve noticed that there are not a lot of defined roles for a PC across the state. A lot of other stuff gets blurred in.”

Most KIs reported that their responsibilities within each domain, if not across domains, are clearly defined. This is primarily a result of good communication and strong coordination between the local health department and county emergency services. 

### 3.3. Routine Collaborations with Other Public Health and Emergency Services Agencies

In order to fulfill their dual roles, KIs consistently reported collaborating with the county health director, emergency management director, local law enforcement, fire department, emergency medical services, other emergency response partners, and regional hospitals. Some KIs are actually hospital-based emergency managers, and similarly report collaborations with the local health department and emergency management services:

“You name it, we work with them. It’s to the point where I have a key to the health department and 24 h access. We’re in constant contact.”

All counties have meetings at least quarterly with some or all of their collaborators. Most report quarterly meetings with their Local Emergency Planning Committees (LEPCs), with additional monthly meetings among local state and preparedness partners as deemed necessary. See Appendix Table A1 for a list of partner organizations and resources identified by each KI. 

### 3.4. Perceived Motivations for Merging the Positions

Different counties had different motivations for the merger. KIs from two counties reported that the merger was a matter of convenience and efficiency. The overlapping responsibilities between emergency management and public health preparedness, with respect to developing preparedness and response plans and conducting training exercises, were conducive to a shared role. The KI from 1 county reported that the need to develop a pandemic influenza plan in 2006 required an additional staff member, and he was hired to help bridge the gap between public health and emergency management to help write the plan. Another KI also reported that the small size of the county made it a good candidate for the dual role. In these two counties, the position was conceived and developed around the specific skill sets of the people recruited for the position. 

In two other counties, however, the merger was necessary. The KI from one county reported a lack of funding in the health department to hire a full-time preparedness coordinator position. The KI from another county reported the need for a full-time employee to serve the county and manage the emergency management and public health preparedness coordinator responsibilities. Prior to the merger, the emergency management and public health preparedness coordinator positions were each only half-time positions. 

### 3.5. Ability to Carry out Responsibilities in the Emergency Management and Public Health Preparedness Domains

Most KIs reported feeling sufficiently knowledgeable to be able to perform both emergency management and public health preparedness roles. This was a result of strong linkages in both domains that allowed them to consult with other public health or emergency management staff to fill knowledge gaps. The KIs from two counties reported wanting more training in public health substantive areas, specifically epidemiology and environmental health, to help them approach public health preparedness topics more effectively. KIs in two other counties reported having a firm grasp on the basics, but would like more training in the nuances of both emergency management and public health preparedness. Because the position is new, standard job requirements and scopes of work have not yet been developed for use across counties. As a result, individuals in this position are often learning on the job:

“It would be a learning process for anybody. There isn’t any school or any class or any one thing that would really prepare you for this position. I knew about PODs (Points of Dispensing) from my emergency management background. But we didn’t have any training on what a POD was, how to operate a POD, what the parts of the POD were. All we were told is when they bring you down here and they hand you all this medicine you’re supposed to dispense it. So I took the POD class and it made everything clearer.... (But) I am still learning this whole LTAR (Local Technical Assistance Review) thing.”

The KI from one county similarly reported that the training exercises in PODs and other topics were helpful to them; however, budget cuts in the last couple years have made it so that many of those new to these positions will not receive critical training. All KIs reported being well-supported in fulfilling their duties in both the emergency management and public health preparedness domains by their supervisors, coworkers, and external partners. Strong linkages and positive relationships between the public health and emergency management departments ensure that individuals in the new position have the time, resources, and training to complete their duties in both capacities. 

### 3.6. State of Public Health Preparedness

Most KIs reported that their emergency preparedness plans were better than they were prior to the merger, or at least will become better in the long run. This is due primarily to the merging and streamlining of public health preparedness and emergency management plans, which recognizes the extensive overlaps between them. The plans are now more cohesive and concise and more easily accessible to a variety of users:

“Needless to say, if you’ve got 40 hours to devote (to preparedness) rather than 20, you’re able to do more.”

One KI expressed doubts that plans would necessarily improve in a county with a larger population (*i.e.*, greater than 200,000), where there may not be sufficient expertise in both subject areas to be able to write preparedness plans to cover such a large jurisdiction. The counties included in this case study who had implemented the shared public health and emergency management, emergency medical services, or hospital preparedness positions had an average population of 71,000 (Range: 38,000–133,000).

### 3.7. Barriers to Implementation of the Merged Position

Each KI identified unique challenges in their jurisdiction to the effective implementation of the merged emergency management and public health preparedness coordinator position. One county reported that funding for salaries and supplies is split between the emergency management and public health preparedness budgets, and that it is sometimes difficult to prioritize spending for supplies and resources that are critical for emergency management. Another county reported that funding for public health is much more restricted and limited than for emergency management, and that the requirements to receive public health preparedness funding from the CDC are more stringent. In the opinion of some KIs, the time spent fulfilling these requirements may not worth the sum of money received:

“Public health is very specific on what you can spend your funds on. They would buy things that I don’t know would benefit us.”

One KI reported that their county has not experienced any major emergencies recently. The KI worried that the resulting complacency might make it harder to prepare for and respond to emergencies. Without the threat of an emergency, there is less pressure or incentive to focus on preparedness:

“The biggest barrier is, knock on wood, we haven’t had much in the way of disaster related events. After 9/11 people got saturated with it for a while, but people have gotten a little more complacent.”

One KI described a large gap between public health and emergency management at the state level, in which the N.C. Division of Public Health may not reach out to N.C. Emergency Management for support, meaning that a lot of efforts are duplicated. These communication issues may negatively affect operations at the local level if state agencies request duplicative deliverables:

“Public health should not be requiring our auxiliary communications folks to prove that they test their systems on a regular basis. Doing the job is not a problem, satisfying the needs of somebody else in (Washington, DC) or Raleigh (N.C.) is the problem.”

Another KI did not report personal barriers, but speculated that uneven funding and timing split between the emergency management and public health preparedness domains may pose challenges in counties that are more siloed and have a hard time coordinating efforts across departments. One or the other domain will receive less support. 

## 4. Conclusions

In the six counties in North Carolina in which this model has been implemented, it has contributed to the improved quality of public health preparedness and emergency operations plans and streamlined processes related to both emergency management and public health preparedness. It has also helped to cut costs and eliminate redundancy of responsibilities. Remaining challenges include dividing funding and responsibilities for each domain appropriately to ensure that neither side is neglected. Overall, the model appears to function well in a variety of counties in North Carolina, and all the KIs included in this study would recommend the model of the shared emergency management and public health preparedness position for other counties, provided that they meet the following conditions:

- The county must be small enough for the responsibilities of emergency management and public health preparedness to be fulfilled by one person;
- The individual in the role has sufficient expertise in emergency management and public health to be able to perform effectively;
- The county emergency management and public health departments are working towards the same goals, and;
- Funding for the role is split evenly between emergency management and public health preparedness to ensure that both domains are given equal priority.

This study has several limitations. As an exploratory case study with a very small sample size, findings cannot be generalized beyond the counties that participated. Additional research and evaluation studies are needed in order to confirm KI perceptions. Future research could include objective comparisons of the quality of plans written in a jurisdiction where these positions had been merged with one where they had not been merged or observing and evaluating exercises or emergency responses in jurisdictions with both merged and unmerged positions.

## Appendix

**Table A1:** Partnerships and collaborations with local agencies reported by key informants.

Agency	Partner *	Resource ^†^	Neither ^‡^
Local health department director	5	0	1
Local health department staff	6	0	0
Regional offices of the N.C. Office of Public Health Preparedness and Response	5	1	0
Regional hospital-based Public Health Epidemiologists	3	3	0
Office of Public Health Preparedness at the N.C. Division of Public Health	3	3	0
Office of Occupational Epidemiology at the N.C. Division of Public Health	2	4	0
Communicable Disease Branch at the N.C. Division of Public Health	4	2	0
N.C. State Public Health Laboratory	2	4	0
N.C. Department of Environment and Natural Resources	1	4	1
N.C. Department of Agriculture and Consumer Services	2	3	1
Local Office of Emergency Management	6	0	0
N.C. Emergency Management Regional Offices	4	2	0
N.C. Office of Emergency Management	4	2	0
Hazardous Materials Regional Response Teams	2	4	0
State Medical Assistance Teams	5	1	0
Domestic Preparedness Readiness Regional Committees	4	2	0
Local Health Information Teams	2	1	3
Cities Readiness Initiative	0	1	5
Local Emergency Planning Committees	4	1	1
Medical Reserve Corps	3	0	3
State Animal Response Teams	1	2	3
N.C. Veterinary Response Corps	1	3	2
Intrastate Crisis Communication Enhancement Network	1	4	1
Search and Rescue Task Force Teams	1	4	1
Local Emergency Medical Services	6	0	0
Emergency Medical Services Regional Advisory Committee	5	0	1
N.C. Office Emergency Medical Services	3	2	1
Local or regional hospitals and healthcare providers	6	0	0
Hospital infection control nurses	5	0	1
Hospital safety officers	3	1	2
Local or state law enforcement	6	0	0
Local fire services	5	1	0
Military preparedness organizations	1	1	4
School safety coordinators	3	1	2
School administration	5	1	0
Correctional facilities	0	1	5
U.S. or State Forest Service	3	2	1
Airport or customs officers	1	1	4
N.C. Ports	1	0	5
County commissioners	5	0	1
Department of Veterans Affairs	2	2	2
American Red Cross	6	0	0
Social work agencies	5	1	0
Mental health and substance abuse agencies	2	2	2
Religious organizations	2	4	0
Academic institutions	4	2	0
Private businesses	4	2	0

Notes: ***** Partner: An agency with whom the KI worked regularly to carry out job duties. **^†^** Resource: An agency on whom the KI could rely for information or assistance with a project, but with no formal relationship. **^‡^** Neither: An agency that either does not exist in the KI’s county, or with which he or she did not associate.
